# Comprehensive dataset on macro-porous PVDF flat sheet membranes for membrane distillation: Materials characteristics, morphology and performance data

**DOI:** 10.1016/j.dib.2026.112588

**Published:** 2026-02-10

**Authors:** Sven Johann Bohr, Ioannis Tournis, Sascha Fahlberg, Benjamin Schneider, Marius Melzer, Andreas Sapalidis, Evangelos P. Favvas, Stéphan Barbe

**Affiliations:** a Faculty of Applied Natural Sciences, TH Köln - Cologne University of Applied Sciences, Campusplatz 1, 51379 Leverkusen, Germany; b Department of Technical Chemistry II, University of Duisburg-Essen, Universitätsstraße 7, 45141, Essen, Germany; c Membranes and Materials for Environmental Separations Laboratory, Institute of Nanoscience and Nanotechnology, National Center for Scientific Research “Demokritos”, Patriarchou Grigoriou E & 27, Neapoleos str, Agia Paraskevi, 153 41, Athens, Greece; d Sartorius-Stedim Biotech GmbH, August-Spindler-Straße 11, 37079, Göttingen, Germany

**Keywords:** Design of experiment, PVDF, NMP, VIPS, NIPS, VNIPS, Dissolution temperature, Membrane distillation

## Abstract

This data article presents a structured dataset from the transfer of a proven PVDF hollow-fiber formulation to macro-porous PVDF flat sheet membranes via a vapor-assisted non-solvent induced phase separation (VNIPS) process designed for membrane distillation (MD). The study uses N-methyl-2-pyrrolidone (NMP) as a solvent and water as a non-solvent. A face-centered composite design (33 membranes) was implemented to efficiently sample six controllable factors at three levels each: polymer content (16–20 wt %), solvation temperature (30–60 °C), wet casting thickness (300–500 µm), vapor-induced phase separation (VIPS) conditioning time (60–360 s at fixed 90 % RH), coagulation bath temperature (25–50 °C), and bath solvent content (0–50 wt %). For each treatment, one 100 × 200 mm membrane was cast on a lab-scale line (dope dispense + doctor blade → VIPS tunnel with controlled humidity/airflow → NIPS bath), then transferred to polyester fleece and dried at 40 °C for 24 h. Materials comprised PVDF (Mw ≈ 534 kDa), N-methyl-2-pyrrolidone (NMP, 99.5 %), and RO water.

The dataset includes: (i) full experimental design with factor settings for all 33 runs; (ii) raw and processed characterization results—mean thickness, water contact angle, liquid entry pressure, porosity, and permeate flux—for each membrane; (iii) linear regression models linking process factors to responses, constructed by retaining only coefficients with *p* < 0.05 and summarized by goodness-of-fit (R²: 67–94 % across endpoints); and (iv) scanning electron microscopy (SEM) micrographs of the top surface, bottom surface, and cross-section of each membrane, capturing morphological changes across the design space.

The accompanying tables also report the materials list and an optimization scenario (target-value approach) that returns factor settings maximizing wetting resistance and flux within the explored design bounds. These data enable reuse for multiple purposes: reproducing and extending VNIPS DoE studies; meta-analysis of factor–response relationships in phase inversion casting; benchmarking inverse design, response-surface, or machine-learning models; informing scale-up constraints and uncertainty analyses; and guiding MD membrane pre-screening under alternative objective functions or constraints.

Specifications Table

**Table:** 

Subject	Engineering & Materials science
Specific subject area	Design of experiment assisted porous polymer membrane fabrication optimization
Type of data	Table (.xlsx format), Raw. Image (.tif format), Raw.
Data collection	Data were collected by micrometer caliper, contact angle measurement, dead end filtration, porometry, lab-scale DCMD, SEM. Data were analyzed by Minitab Statistical Software v20.3.
Data source location	Country: Greece and Germany.
Data accessibility	Repository name: OSF Data identification number: 10.17605/OSF.IO/WB8CV Direct URL to data: https://doi.org/10.17605/OSF.IO/WB8CV
Related research article	none

## 1 Value of the Data

• **Why these data are valuable.** They provide a complete, design-of-experiments (DoE) [1] dataset for vapor-assisted nonsolvent induced phase separation (VNIPS) [2] preparation of PVDF flat-sheet membranes, including controlled factor settings (polymer content, solvation temperature, casting thickness, VIPS time, coagulation temperature, bath solvent content), raw and processed measurements (thickness, water contact angle, liquid entry pressure, porosity, permeate flux), and SEM micrographs (top, bottom, cross-section) for every run. The combination of process variables, quantitative responses, and morphology images in one package is rarely available in open form.
• **Why these data were generated.** To create a transparent, reproducible record of a VNIPS/NIPS casting campaign that systematically samples a practical process window using a face-centered composite design, with standardized materials, operating steps, and characterization readouts. The intent is to make the experimental matrix, accompanying protocols, and analysis-ready tables accessible for verification, comparison, and downstream method development.
• **How others can reuse them (modeling and inference pipelines).** Researchers can benchmark response-surface methods, regularized regression, and machine-learning models for experiment planning or inverse design; perform sensitivity and variance-partitioning studies; or validate uncertainty-aware optimizers using the provided factor space, response tables, and baseline linear models.
• **How others can reuse them (morphology and imaging).** The paired SEM micrographs (top/bottom/cross-section per treatment) support quantitative image analysis, automated segmentation, and morphology–property feature extraction; they can train or validate computer-vision workflows for pore architecture metrics, tortuosity proxies, or cross-sectional texture descriptors.
• **How others can reuse them (replication, extension, and education).** The DoE matrix and operating ranges enable straightforward replication on different equipment, extension to new factor levels or additives, and meta-analyses across labs. The dataset also suits teaching modules on experimental design, membrane fabrication workflows, and data stewardship, including exercises that connect process settings to standardized characterization outputs.

## 2 Background

This dataset was compiled by the authors during a controlled campaign to document the transfer of a proven PVDF membrane formulation to macro-porous flat sheets produced by vapor-assisted non-solvent induced phase separation (VNIPS). The motivation was to record, in a single resource, the full experimental matrix, operating conditions, and standardized readouts associated with a design-of-experiments (DoE) study relevant to membrane distillation. Methodologically, a face-centered composite design was used to sample a practical process window across six factors: polymer content, solvation temperature, wet casting thickness, vapor-induced conditioning time at fixed relative humidity, coagulation bath temperature, and bath solvent content. Each treatment consisted of casting on a lab line (dope dispense and doctor blade → controlled-humidity tunnel → coagulation bath), transfer to a polyester support, and low-temperature drying. Materials included PVDF in N-methyl-2-pyrrolidone with water as non-solvent. Standardized characterization comprised thickness, water contact angle, liquid entry pressure, porosity, permeate flux, and scanning electron microscopy of the top surface, bottom surface, and cross-section. The dataset consolidates factor settings, raw and processed measurements, and imaging to provide a complete record of conditions and outcomes within the explored design space.

## 3 Data Description

The dataset of the linked repository is organized into (i) tabulated process and performance data, contained in a single Excel workbook, and (ii) imaging data, organized in subfolders with scanning electron microscopy (SEM) micrographs for each membrane sample.

### 3.1 Tabulated data

All numerical and categorical data are stored in the Excel file “Experimental parameters and results.xlsx”. This file contains several worksheets. The following table summarizes structure and the content of the dataset. Table 1

**Table 1:** Contents and structure of the Excel workbook. Each Sample ID in the “DoE factors & results” sheet corresponds directly to one numbered subfolder in the SEM directory.

Sheet name	What it contains
**DoE factors & results**	Full experimental design with **metadata, experimental factors**, and **measured responses** in raw form of each run. • **Metadata:** s*tandard order*, r*un order*, s*ample ID*. • **Experimental factors** (in uncoded units)**:** p*olymer content* (wt. %), *solvation temperature* ( °C), *casting thickness* (µm), *VIPS conditioning time* (s), *coagulation bath temperature* ( °C), and *bath solvent content* (wt. %). • **Measured responses:** mean *dry membrane thickness* (µm), *95* *% confidence interval (CI) dry membrane thickness* (µm), *mean static water contact angle (WCA)* (°), *95* *% CI WCA* (°), *liquid entry pressure* (LEP) (bar), *porosity* ( %), and *MD permeate flux* (kg m⁻² h⁻¹). An additional response, *Salt rejection* ( %), derived from the ratio of *feed conductivity* (µS/cm) to *permeate conductivity* (µS/cm), is included but not part of the RSM analyses.
**Dope batch formulation**	Composition of each polymer dope batch, with the *batch size* (g), the *PVDF* and *NMP mass fractions* (wt. %), and the *component masses* (g) and *volumes* (mL). The *Dope batch ID* follows the pattern **xx-yy**, where xx = mass fraction of polymer (wt. %) and yy = dissolution temperature ( °C).
**Coagulation bath formulation**	Composition of each coagulation bath, with the *bath size* (g), the *water* and *NMP mass fractions* (wt. %), and the *component masses* (g) and *volumes* (mL). The *Bath ID* follows the pattern **CB xx**, where xx = mass fraction of solvent (NMP) ( %).
**h_dry RSM analysis**	The response surface analysis (RSM) of *mean dry membrane thickness* (h_dry) (µm) includes: • **Model terms:** Specifies the terms used in the model. • **Removed terms:** Lists the terms that were removed from the model. • **Number of unused experimental runs:** Number of runs not used by the model. • **Method:** Specifies method for identifying a useful subset of model terms. • **Coefficient categories:** coded *coefficients* of each *term* with standard error *(SE Coef)*, 95 % confidence interval *(95* *% CI), T-value, P-value*, and variance inflation factor *(VIF)*. • **Model summary metrics:** residual standard deviation *(S)*, coefficient of determination *(R²)*, adjusted R² *(R² adj)*, predicted R² *(R² pred)*, predicted residual sum of squares *(PRESS)*, corrected Akaike information criterion *(AICc)*, and Bayesian information criterion *(BIC)*. • **Analysis of Variance (ANOVA):** *source* of the degrees of freedom *(DF)*, sequential sum of squares *(Seq SS)*, adjusted sum of squares *(Adj SS)*, adjusted mean squares *(Adj MS), F-value*, and *P-Value*. • **Regression equation in coded units** • **Observation-level diagnostics:** fitted value *(Fit)*, standard error of fit *(SE Fit), Contribution* ( %), confidence interval *(CI)*, residual *(Resid)*, standardized residual *(Std Resid)*, deleted residual *(Del Resid)*, leverage *(HI)*, Cook’s distance *(Cook’s D)*, and difference in fits *(DFITS)*. • **Regression equation in coded units:** of the *mean dry membrane thickness* (µm). • **Observation-level diagnostics:** *Standard Order*, observed response value mean dry membrane thickness *(h_dry)* (µm), Fitted response value *(Fit)*, Standard error of the fitted value *(SE Fit)*, 95 % confidence interval for the fitted value *(95* *% CI)*, Residual *(Resid)*, Standardized residual *(Std Resid)*, Deleted Studentized residual *(Del Resid)*, Leverage *(HI)*, Cook’s distance *(Cook’s D)*, Difference in fits *(DFITS). R* signifies a large residual.
**WCA RSM analysis**	Same structure as above, for the RSM of *mean water contact angle* (WCA) (°).
**LEP RSM analysis**	Same structure as above, for the RSM of *liquid entry pressure* (LEP) (bar).
**Porosity RSM analysis**	Same structure as above, for the RSM of *porosity* ( %).
**MD flux RSM analysis**	Same structure as above, for the RSM of *MD permeate flux* (MD flux) (kg m⁻² h⁻¹).

### 3.2 SEM micrographs

All SEM images are stored as .tif files in the SEM subfolder, organized by Sample ID. The structure of the filenames indicates sample linkage, replicate, view (LS/BS/QS), and magnification according to the following pattern.

SEM\aa\MM_bbbb_PVDF_SA_c.d-ee_ffffx.tif

• aa – Subfolder label = Sample ID (1 – 42).
• MM_bbbb_PVDF – Internal project and unique sample identifier (bbbb).
• SA_c – Sample-level identifier (*c* = Sample ID).
• d – Replicate number of the same sample.
• ee – Membrane view: LS = top side, BS = bottom side, QS = cross-section.
• ffffx – Magnification (e.g., 1.000x, 8.000x).

Each numeric subfolder contains at least one LS (top surface), one BS (bottom surface), and one QS (cross-section) micrograph, with magnifications typically ranging from 1.000x to 10.000x.

## 4 Experimental Design, Materials and Methods

This section first describes the materials and solution preparation procedures, followed by the DoE-assisted membrane fabrication process and the characterization methods used to generate the dataset.

### 4.1 Materials

Polyvinylidene fluoride (PVDF, CAS 24,937-79–9, M_w_ = 534 kDa, from VWR International, Darmstadt, Germany), 1-Methyl-2-pyrrolidinone (NMP, CAS 872–50–4, 99.5 %, analytical grade, from Fisher Scientific, Schwerte, Germany), and reverse osmosis water (Sartorius, Göttingen, Germany) were used as received and used in the DoE for membrane preparation. All experimental designs, their statistical analyses and modelling were performed using Minitab v. 20.3.

### 4.2 Methods

#### 4.2.1 Solution preparation

One polymer solution was prepared for each combination of polymer content and dissolution temperature. In total 9 solutions were prepared. Each solution was prepared as follows. The appropriate amount of solvent was put in a 3-neck-flask and stirred with an overhead stirrer at 150 rpm. The appropriate amount of PVDF was added. Then the temperature of the oil bath was set to the respective dissolution temperature. The solution was stirred over night for about 10 h.

#### 4.2.2 Membrane preparation

**Fig. 1:**
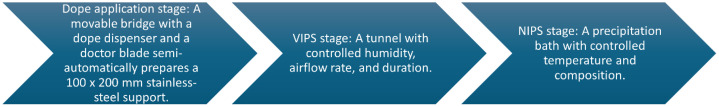
Chevron process of the lab scale flat sheet membrane production line. Left side: dope application; middle section: VIPS stage; right side: NIPS stage.

The DoE-assisted membrane fabrication was conducted at the laboratory of Sartorius Stedim Biotech GmbH on a lab scale flat sheet membrane production line for VIPS + NIPS. Fig. 1 shows a Chevron process of the production line. It consists of a semi-automated dope application onto a 100 × 200 mm stainless-steel support, via a movable bridge that incorporates a dope dispenser and a doctor blade. The support moves via conveyer belt into a VIPS stage with controlled humidity and airflow rate. The conveyer belt is halted in the VIPS stage for the time specified in the DoE. The relative humidity was fixed at 90 % and the airflow velocity was set to 1.3 m/s. Subsequently, the conveyer belt moves the support into a precipitation bath (NIPS stage). The bath composition and temperature were as specified by the DoE. The precipitation duration was 30 min. After the NIPS stage the membranes were removed from the support and placed into a second bath of RO water at room temperature (20 °C) for 30 min to remove any remaining solvent. Subsequently, the membranes were placed on a polyester fleece and dried at 40 °C for 24 h in a forced-air drying oven at atmospheric pressure Fig. 1.

#### 4.2.3 SEM

Membrane samples were prepared for imaging with a Apreo 2 s LoVac (Thermo Fisher) field-emission scanning electron microscope (FE-SEM) as follows. Approximately 5 × 5 mm samples were cut from each membrane. Cross-sections were generated by immersing the membranes in liquid nitrogen and fracturing them immediately to produce clean brittle breaks perpendicular to the surface. The samples were sputter coated with gold. Imaging was performed at accelerating voltages of 2 – 5 kV and working distances of 5 – 10 mm. Micrographs were acquired at 800x to 10.000x magnification for each membrane surface (LS, BS) and cross-section (QS) and exported as .tif files according to the defined naming convention. The membrane thickness measured by SEM is not representative of the membrane’s average thickness due to the sample preparation with liquid nitrogen.

#### 4.2.4 Membrane thickness

The mean thickness of each membrane was determined using a digital micrometer caliper with a resolution of 1 µm. For each sample, the thickness was measured at five different positions across the membrane surface. The mean thickness and the corresponding 95 % confidence interval (CI) were calculated for each membrane.

#### 4.2.5 Membrane liquid entry pressure

The liquid entry pressure (LEP) was measured to evaluate the wetting resistance of the membranes. A dead-end filtration setup filled with water was used for this test. The membrane sample was mounted inside the filtration cell, and the feed-side pressure was increased in 0.1 bar increments, allowing a stabilization time of two minutes after each step. The LEP was defined as the pressure at which transmembrane flux was first observed.

#### 4.2.6 Membrane porosity

The porosity of the membranes was determined using the gravimetric liquid uptake method, which relates the volume of liquid absorbed within the pores to the total volume of the membrane. Prior to measurement, the samples were dried under vacuum at room temperature for 24 h to remove residual moisture and weighed to obtain the dry weight *W_d_*. The membranes were then fully immersed in isopropanol for 24 h to ensure complete wetting of all pores, after which the excess surface liquid was gently removed using lint-free tissue. The wet weight *W_w_* was recorded immediately, and the porosity *ε* was calculated using the following equation:

$$\varepsilon =\frac{W_{w}-W_{d}}{\rho_{L}V}$$

where *ρ_L_* is the density of isopropanol and *V* is the total volume of the membrane sample, determined from its measured area and mean thickness.

#### 4.2.7 Membrane MD flux

The direct contact membrane distillation (DCMD) performance of the membranes was evaluated on a laboratory-scale setup operated in counter-current flow mode. The membrane sample was mounted in a flat-sheet DCMD cell with an active membrane area of 0.0007 m². A 35 g/L NaCl aqueous solution (feed conductivity ≈ 50,000 µS/cm) was circulated on the feed side at a flow rate of 80 mL/min, while deionized water was circulated on the permeate side at a flow rate of 70 mL/min. The feed temperature was maintained at 75 °C, and the permeate temperature at 15 °C, establishing a transmembrane temperature difference of 60 °C. Both streams were recirculated using peristaltic pumps, and their temperatures were controlled via external thermostatic baths. The permeate mass was recorded over time using an analytical balance, and the steady-state flux *J* was calculated according to

$$J=\frac{\Delta m}{A,\Delta t}$$

where *Δ_m_* is the mass of collected permeate, *A* is the active membrane area, and *Δ_t_* is the sampling time.

#### 4.2.8 Membrane salt rejection

The salt rejection of the membranes was determined simultaneously during the DCMD flux measurements by analyzing the electrical conductivities of the feed and permeate streams. The conductivity of the feed *C_f_* and permeate *C_p_* were measured using calibrated conductivity probes at the beginning and end of each experiment to ensure stable readings. The salt rejection *R* was calculated according to

$$R=\left(1-\frac{C_{p}}{C_{f}}\right)\times \;100$$

#### 4.2.9 Membrane maximum pore radius

The maximum pore radius was determined using the bubble point technique in accordance with ASTM F316. This method is based on the principle that the pressure required for the first gas bubble to pass through a membrane wetted with a liquid of known surface tension is inversely proportional to the size of the largest pore. Prior to measurement, the membranes were fully wetted with isopropanol, ensuring complete pore filling. The wetted membrane was mounted in a dead-end filtration cell, and nitrogen gas pressure was gradually increased in small increments until the appearance of the first continuous gas bubbles on the permeate side. The corresponding pressure was recorded as the bubble point pressure *P*, from which the maximum pore radius was calculated using the Laplace equation $r_{max}=\frac{2\gamma \cos\theta }{P}$, where *γ* is the surface tension of the wetting liquid and θ is the contact angle between the liquid and the pore wall.

#### 4.2.10 Membrane water contact angle

The static water contact angle (WCA) was measured using a DSA-30 Drop Shape Analyzer (Krüss GmbH. For each sample, three separate specimens were analyzed, and ten measurements were taken from different surface locations at room temperature (20 °C). The mean contact angle and the 95 % CI were then calculated for each sample. Deionized water (4 µL) served as the sessile drop, and the affinity of the drop with the surface was determined using the ellipse (tangent) fitting method.

## Limitations

A few limitations were encountered during data collection and curation. In total, five experimental runs resulted in membranes that were too brittle to undergo all characterization procedures. These specimens exhibited insufficient mechanical stability and failed during the DCMD flux tests, preventing the acquisition of complete performance data. Consequently, the absence of MD flux and related response values for these five samples reduced the total number of valid data points available for modeling, thereby diminishing the statistical robustness and predictive validity of the RSM analysis. Additionally, it was later observed that the polymer dissolution time, in addition to the controlled polymer dissolution temperature, had a significant but unaccounted influence on membrane appearance and performance. This parameter was not explicitly controlled or recorded within the DoE. Instead, the preparation criterion was that the polymer solutions appeared visually homogeneous and transparent. This uncontrolled variation may have contributed additional experimental noise. Overall, while the dataset remains internally consistent with respect to the defined DoE factors, the missing performance data and unmonitored dissolution time represent limitations that slightly reduce the dataset’s completeness and statistical confidence. Furthermore, the membranes with the sample IDs 38, 39, and 41 were not subjected to SEM analysis, and sample ID 8 has only SEM micrographs of the membrane cross-section.

## Ethics Statement

The authors confirm compliance with the ethical requirements for publication in *Data in Brief* as stated in Elsevier’s “Policies and Guidelines for Researchers.” This work involves only laboratory-generated materials and process data; no human subjects, animal experiments, or social media data were included. Ethical approvals and informed consent were therefore not applicable. All data were collected and reported in accordance with responsible research and publication standards.

## Credit Author Statement

**Sven Johann Bohr:** Conceptualization, Methodology, Resources, Investigation, Formal analysis, Validation, Data curation, Visualization, Writing – original draft, Project administration; **Ioannis Tournis:** Investigation, Formal analysis, Visualization, Data curation; **Sascha Fahlberg:** Investigation; **Benjamin Schneider:** Project administration, Supervision; **Marius Melzer:** Investigation; **Andreas Sapalidis:** Funding acquisition, Project administration, Supervision, Writing - review and editing; **Evangelos P. Favvas:** Funding acquisition, Supervision, Writing - review and editing; **Stéphan Barbe:** Funding acquisition, Project administration, Supervision, Writing – review and editing.

## Data Availability

OSFComprehensive dataset on macro-porous PVDF flat sheet membranes for membrane distillation: materials characteristics, morphology and performance data (Original data).
